# Progress in the Enhanced Use of Electronic Medical Records: Data From the Ontario Experience

**DOI:** 10.2196/medinform.6928

**Published:** 2017-02-22

**Authors:** Mavis Jones, Chad Koziel, Darren Larsen, Plumaletta Berry, Elena Kubatka-Willms

**Affiliations:** ^1^ OntarioMD Toronto, ON Canada; ^2^ Formerly with OntarioMD Toronto, ON Canada; ^3^ Formerly with the Ontario Medical Association Toronto, ON Canada

**Keywords:** electronic medical record, quality of health care, primary care

## Abstract

**Background:**

This paper describes a change management strategy, including a self-assessment survey tool and electronic medical record (EMR) maturity model (EMM), developed to support the adoption and implementation of EMRs among community-based physicians in the province of Ontario, Canada.

**Objective:**

The aim of our study was to present an analysis of progress in EMR use in the province of Ontario based on data from surveys completed by over 4000 EMR users.

**Methods:**

The EMM and the EMR progress report (EPR) survey tool clarify levels of capability and expected benefits of improved use. Maturity is assessed on a 6-point scale (0-5) for 25 functions, across 7 functional areas, ranging from basic to more advanced. A total of 4214 clinicians completed EPR surveys between April 2013 and March 2016. Univariate and multivariate descriptive statistics were calculated to describe the survey results.

**Results:**

Physicians reported continual improvement over years of use, perceiving that the longer they used their EMR, the better patient care they provided. Those with at least two years of experience reported the greatest progress.

**Conclusions:**

From our analyses at this stage we identified: (1) a direct correlation between years of EMR use and EMR maturity as measured in our model, (2) a similar positive correlation between years of EMR use and the perception that these systems improve clinical care in at least four patient-centered areas, and (3) evidence of ongoing improvement even in advanced years of use. Future analyses will be supplemented by qualitative and quantitative data collected from field staff engagements as part of the new EMR practice enhancement program (EPEP).

## Introduction

Electronic medical records (EMRs) have significant potential to support quality patient care for community-based practices through the following multiple functions: appointment scheduling, practice billing, communication and messaging, encounter documentation, data quality and nomenclature consistency, document management, results management, referral and consultation tracking, prevention and screening, complex care or chronic disease management.

EMR use across Canada has increased steadily over the past few years with growing awareness of practice-level benefits. In 2014, EMR use increased by 53%, with 77% of primary care physicians using EMRs—up from 24% in 2007 [1,2]. In Ontario, over 13,000 physicians, representing an adoption rate of 71% of family doctors and 55% of community-based specialists, have EMRs in their practices. The challenge now is to move beyond the basic use of EMRs to a more advanced use in practices. EMRs can facilitate the collection of population health data for analytics, planning, and delivery, and evidence is also accumulating on financial benefits. In the United States, the case for promoting EMR “meaningful use” via the 2009 HITECH Act [3,4] was supported by early analyses suggesting reduced billing errors at the practice level [5] and an anticipated US $81 billion in annual savings that could eventually double through technology-enabled improvements to prevention, management of chronic disease, and subsequent social benefits [6]. Similarly, a Canada Health Infoway study estimated that annually, nationwide EMR use resulted in workflow savings valued at Can $177 million, reduction in duplicate testing and adverse drug events valued at Can $123 million, and emerging benefits from chronic disease management and preventive care (for example increases in vaccination rates associated with EMR-generated reminders) [7].

Despite anticipated benefits, barriers to achieving optimal EMR use prevent us from fully understanding the impact EMR use might have on upstream processes and downstream outcomes of care [8]. A systematic review by Boonstra and Broekhuis identifies 8 interrelated categories of barriers, including financial, technical, time, psychological, social, and legal. Two additional barriers—organizational and change—should be paid special attention, the authors argue, as they mediate the effects of the others and thereby most directly influence a project’s success [9]. There is indeed a growing literature on EMR implementation as a complex change project [10-13] targeting factors related to cost, time, technical issues, or resistance (fear of change, doubt the investment of resources will be worth it, and so on) [11,12,14-17] . EMR adoption and use operates in a complex adaptive system, highly sensitive to shifting politics and public policy. Larger, well-resourced physician practices and hospitals may be equipped to manage change associated with EMR adoption; others, however, may struggle without help. It is critical that barriers in all types of practices be systematically addressed [7,18] . Here, we describe OntarioMD’s approach to supporting community-based physicians in the adoption and optimization of EMRs and analysis from self-assessment surveys of over 4000 EMR users.

## Methods

### Change Management Approach and Maturity Model

Recognizing that mature use does not necessarily result from the installation of hardware and software in clinics, OntarioMD (a subsidiary of the Ontario Medical Association, with funding from the Ontario Ministry of Health and Long Term Care) applies a practice level change management strategy to address variability in physician EMR use. Modeled on the awareness, desire, knowledge, ability, and reinforcement (ADKAR) framework (Prosci) [19], this approach identifies factors that facilitate or obstruct a user’s capability to adapt to change, generate new knowledge, acquire skills, improve performance, and sustain momentum (Table 1). Multidisciplinary support is available from EMR vendors, peer leaders (experienced EMR users—physicians, clinic managers, and nurses), and the EMR practice enhancement program (EPEP). EPEP provides intensive assistance in the form practice advisors who conduct on-site assessments of current use, identify hidden gaps, and develop action plans to help physicians optimize their EMR use.

**Table 1:** OntarioMD’s change management approach.

ADKAR^a^ elements	OntarioMD supports
Awareness of the need for change	Partnerships and support from the Ontario Medical Association and Ontario government drive awareness of the need and support status as a trusted advisor
Desire to support and participate in the change	Peer leaders validate the practice benefits of EMR^b^ use; self-assessment identifies priority areas
Knowledge of how to change	Practice advisors, peer leaders, and vendors instruct and advise on EMR use in the implementation phase
Ability to implement required skills and behaviours	Practice advisors, peer leaders and vendors support changes in practice workflow and specific functionalities
Reinforcement to sustain the change	Support from practice advisors, peer leaders and vendors continues; EPEP^c^ provides hands-on support to plan concrete actions towards enhanced use

^a^ADKAR: awareness, desire, knowledge, ability, and reinforcement.

^b^EMR: electronic medical record.

^c^EPEP: EMR practice enhancement program.

To anchor this work, OntarioMD developed the EMR maturity model (EMM) and the EMR progress report (EPR) survey tool. Influenced by existing robust models [20-22], the EMM provides a basis for understanding differences in levels of EMR use and the benefits that can be expected with mature use. In the EMM:

- Each key measure is identified as a practice aspect where EMR solutions can have a significant impact, relevant to performance assessment at practice and population levels.
- Each measure can be assessed independently across the 6 maturity levels from 0 to 5, where 0 is paper-based.
- Each level of maturity builds upon the functionality or maturity state of the preceding level.
- EMR capability starts at level 1 based on the specification offerings and requirements of OntarioMD’s EMR adoption program assuming adoption of that aspect of the EMR software.
- Levels 0 to 3 are within scope for an average community-based practice. Levels 4 and 5 mostly reflect potential capabilities (eg, population health) and connectivity, not available in all contexts.

**Figure 1:**
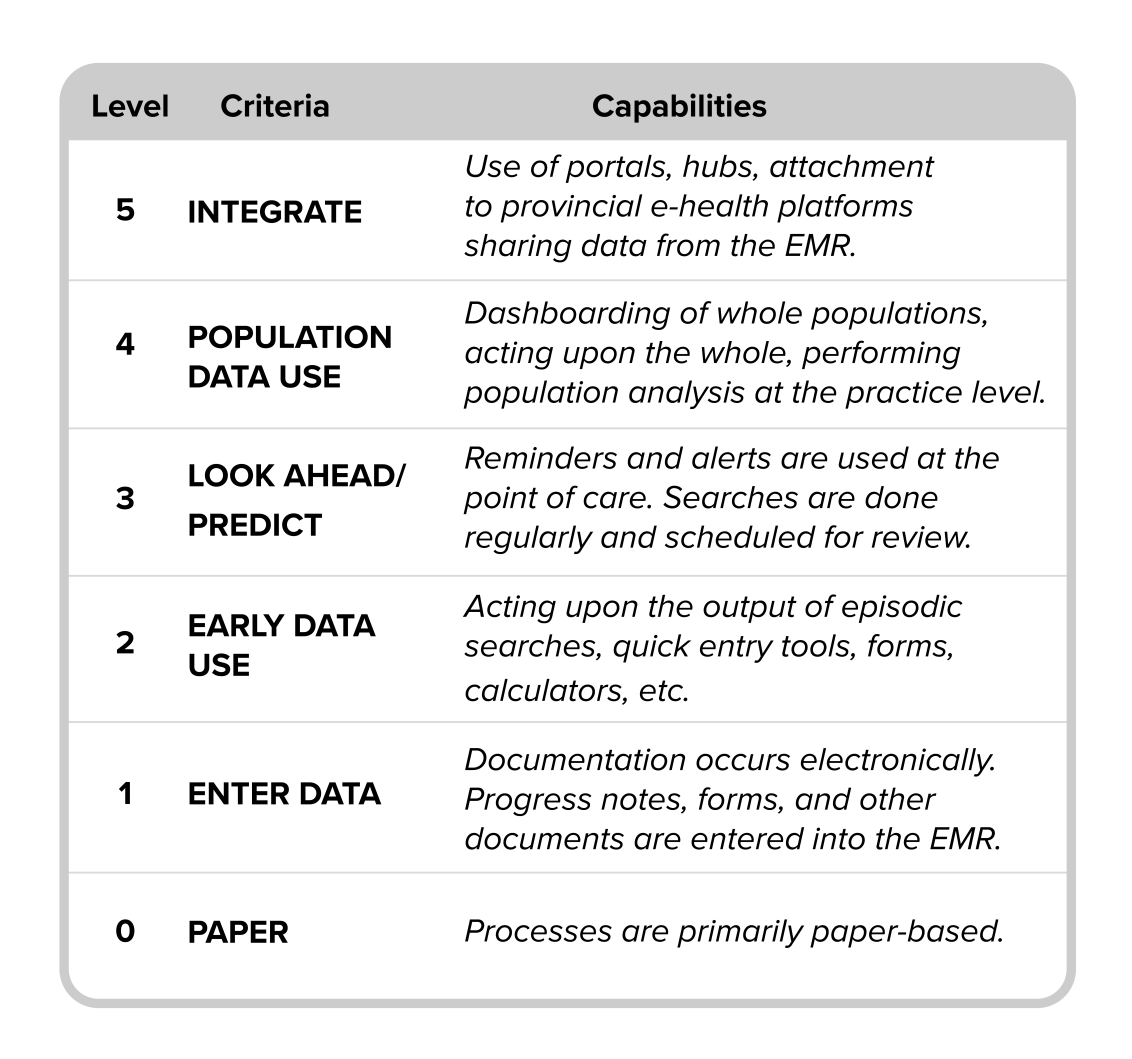
OntarioMD’s electronic medical record maturity model.

Use of these tools provides insight into the reasons for variability in maturity across practices. Figure 1 shows the EMM, as updated in 2016 to coincide with the retirement of the EPR survey in favor of a new tool, with which we are now collecting data for future progress reports (more information on these updates can be found at ontariomd.ca or by contacting the authors). As originally conceived, the EPR allowed for assessment of against the 6 maturity levels (0–5), for 25 EMR functions across 7 functional areas, ranging from basic to more advanced (Table 2).

**Table 2:** Functional areas and corresponding functions mapped on the original electronic medical record (EMR) maturity model (EMM).

Functional area	Functions
Practice management	Appointment scheduling
	Practice billing
	Communication and coordination
	Business continuity planning
Information management	Registration information
	Encounter documentation
	Data quality management
	Nomenclature consistency
	Document management
	Privacy and security
Patient results management	Laboratory results
	Diagnostic image reports
	Hospital summary information
	Referrals and consults tracking
Diagnosis support	Patient assessment tools
	Preventive or follow-up care
	Evidence-based resources
Treatment planning support	Care planning and coordination
	Medication management
	Complex care or chronic disease management
Patient engagement and communication	Patient education
	Self-care or comanagement
Evaluation and monitoring	Health quality indicators
	Health outcome measures
	Public health reporting

### Survey Instrument

**Figure 2:**
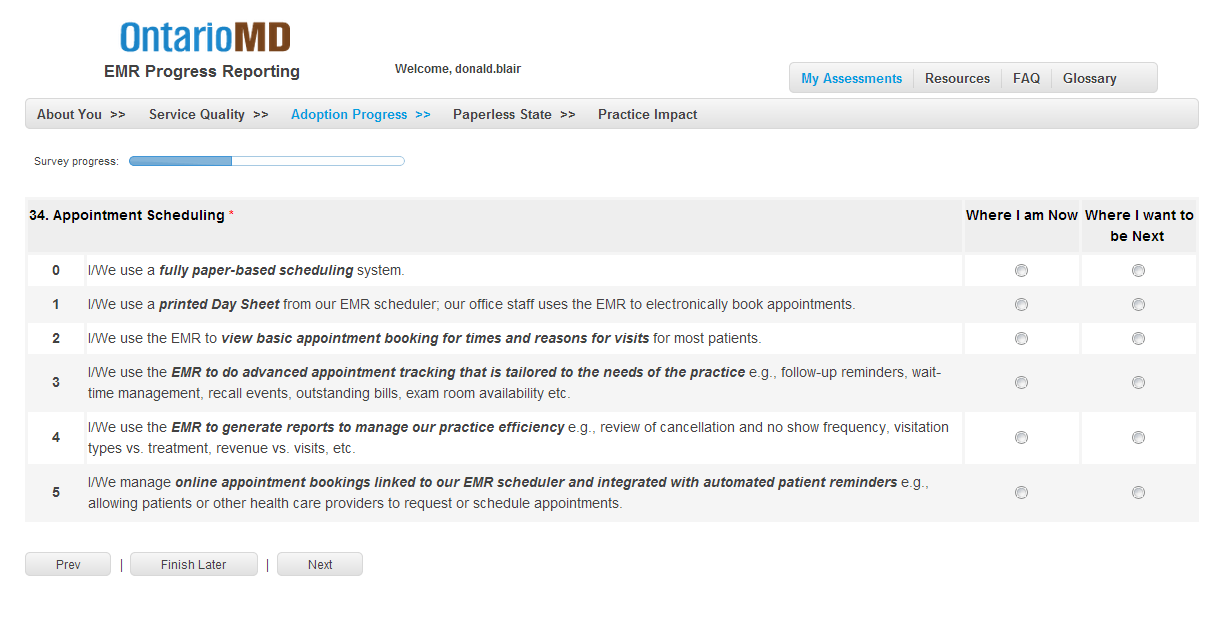
Sample electronic progress report question.

Data presented here were gathered via the EPR, the survey instrument in effect until summer 2016. The EPR was a Web-based self-assessment tool designed to help community-based family physicians and specialists enrolled in OntarioMD’s EMR adoption program identify their current skill level and track progress over time. The survey tool was developed based on the review of the evidence on best practices and evaluation of EMR adoptions within the primary care environment (eg, Health Care Information and Management Systems Society). It was face-validated by the 30 (at that time) members of OntarioMD’s peer leader program, who then pilot-tested the survey among their clinical associates. The tool was subsequently refined and was launched province wide in August 2013. Figure 2 shows a sample screenshot of a question in the EPR.

Since that time, with physicians across the province moving to mature use, both the EMM and survey were updated to reflect the evolving realities of practice across 3 broad functional areas associated with quality patient-centered care: practice management, information management, and diagnosis and treatment support. The EMR progress assessment (EPA), launched in summer 2016, is a more concise instrument for assessing maturity; but, as they essentially measure the same thing, data from the EPR and EPA have been blended to support longitudinal analysis. With the former EPR and now the EPA, physicians have immediate access to their own data on several measures and can compare their performance with the average of physicians surveyed across the province or those in the same practice type (eg, solo or group). In the clinical environment, the EPR tool facilitates benchmarking, gap analysis, customized goal setting, and improvement projects.

### Data and Analysis

Of the 11,650 participants enrolled in the adoption program, 4214 completed at least one EPR. Analyses were run on the most recent EPR completed by each participant (some completed more than 1 EPR between April 2013 and March 2016, for annual assessments). Univariate and multivariate descriptive statistics were calculated to summarize responses, whereas multivariate linear regressions were developed to describe correlations and adjust for the competing effects of variables. Respondents were analyzed using the parameters “type of physician” (family physician or specialist) and “number of years of EMR use” (<2, 2-4, 4-6, >6). Of the 4214 who completed an EPR, almost half were between ages 45 and 64 years (2078); 1776 were 44 years or younger, and 360 were 65 years or older. Surveys were completed by clinicians province wide, across its range of population demographics and densities (urban, suburban, rural, or remote). Information was not collected on physicians’ gender.

### Limitations

The data analyzed here were subject to the following limitations:

- Financial incentives: EMR adoption funding agreements granted physicians a payment upon completing an EPR.
- Self-report: All data is self-reported, thus representing clinicians’ perceptions of improvement rather than measurable improvements against health quality indicators (not standardized in Ontario at the time of launch).
- Technological limitations: Levels 4 and 5 (integrated care and population health impacts) may not be attainable on some measures due to interoperability issues, lack of availability in connected provincial assets, and EMR product-specific limitations.
- Pace of progress: Health information technologies are rapidly evolving and may quickly render the findings presented here outdated. Regular updates could address this problem.
- Variation across areas of specialization: Enrollment in OntarioMD’s funding program was originally only open to family physicians; a limited number of specialists were included in 2009 and both cohorts have grown steadily since then. Rate of enrollment varies significantly across specialties; however, for reasons that are beyond the scope of our analysis, family physicians still comprise the majority of survey respondents.
- Generalizability concerns: The functional areas defined in the tool are broadly applicable—certainly in the Canadian context—as a means to establish within-practice baselines against which progress can be measured. However, caution should be exercised in using these tools to compare or benchmark maturity across regions in a larger geographic area. Adjustment should be made for factors that could influence maturity, such as infrastructural and demographic variations.

## Results

### Overall

**Figure 3:**
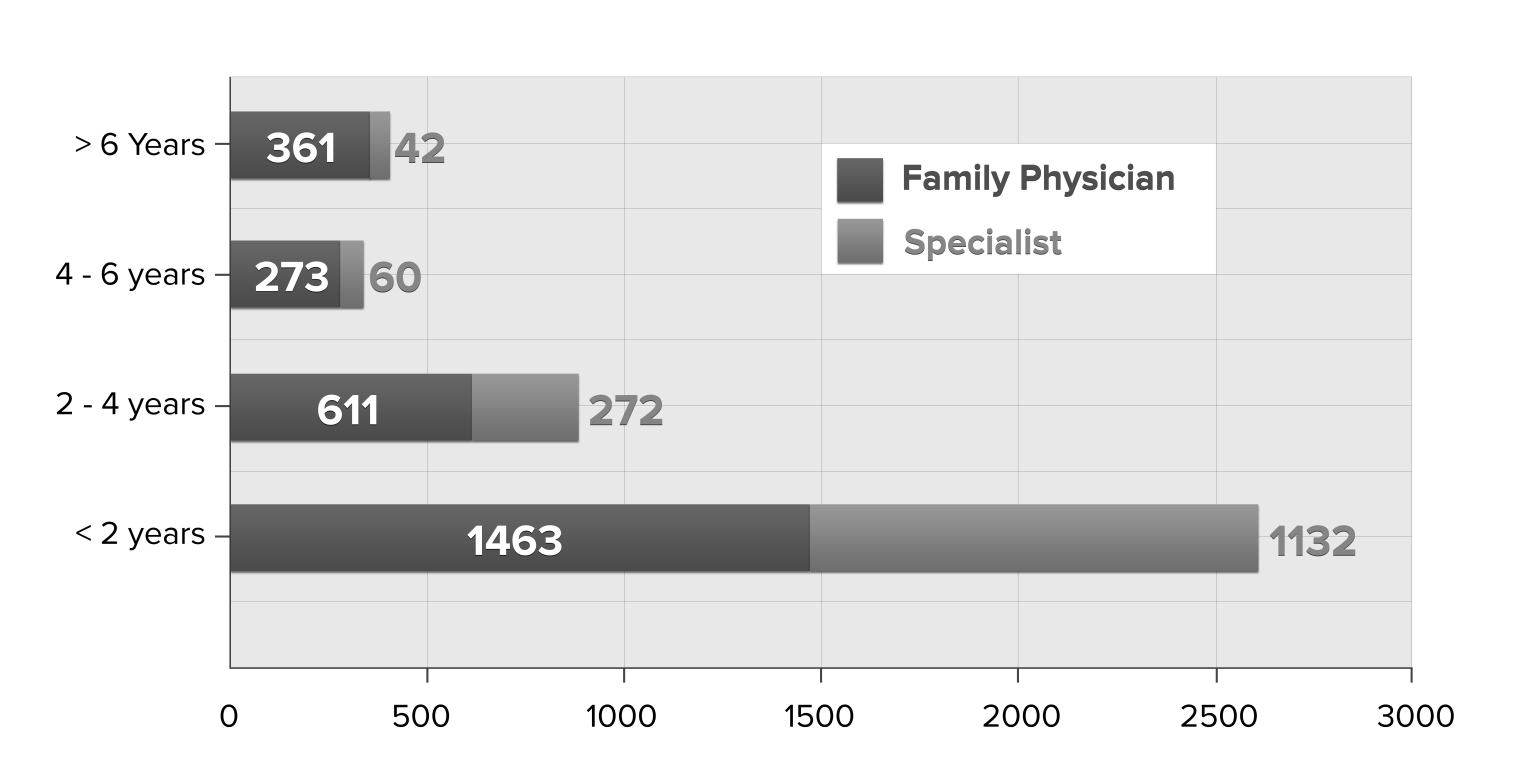
Breakdown of respondents by years of use (N=4214).

Figure 3 shows the breakdown of respondents by type of practitioner (family practice and specialist) and years of EMR use (time since go-live date). The majority of responses were completed within 2 years of adopting a new EMR.

### Self-Reported EMR Maturity by Years of Use

Ideally mature EMR use improves over time. Physicians generally need a minimum of 2 to 3 years of EMR data collection to ensure that their records are adequately populated to support advanced functions, for example, monitoring their patient population to identify those due for preventive screening. 73.94% (2426/3281) of physicians indicated that they were primarily paperless and 25.51% (837/3281) reported using both paper and electronic charts. Comparing data from this sample (all of whom have at least adopted an EMR) with the general population polled in the National Physician Survey [1,2] and bearing in mind semantic nuances in the questions asked, we still saw a steady shift from primarily paper to primary electronic charts between 2007 and 2014 (Table 3). Further, the percentage of Ontario physicians reporting using electronic charts in 2014 was among the highest in the country (tied with BC and Saskatchewan, and second only to Alberta).

**Table 3:** Extent of electronic versus paper-based workflow in physician practices, 2007 and 2014. (source: National Physician Survey; responding samples have been weighted to represent the population size. See www.nationalphysiciansurvey.ca)

Workflow description	Ontario only, 2007	Ontario only, 2014	National, 2007	National, 2014
Total n/N	2286/20,267	3883/26,238	7038/55,398	9711/68,177
Paper charts only	54.8%	16.6%	57.9%	21.3%
Combination of paper and electronic charts	29.8%	48.4%	26.1%	49.3%
Electronic charts instead of paper charts	9.9%	34.9%	9.8%	29.4%

**Figure 4:**
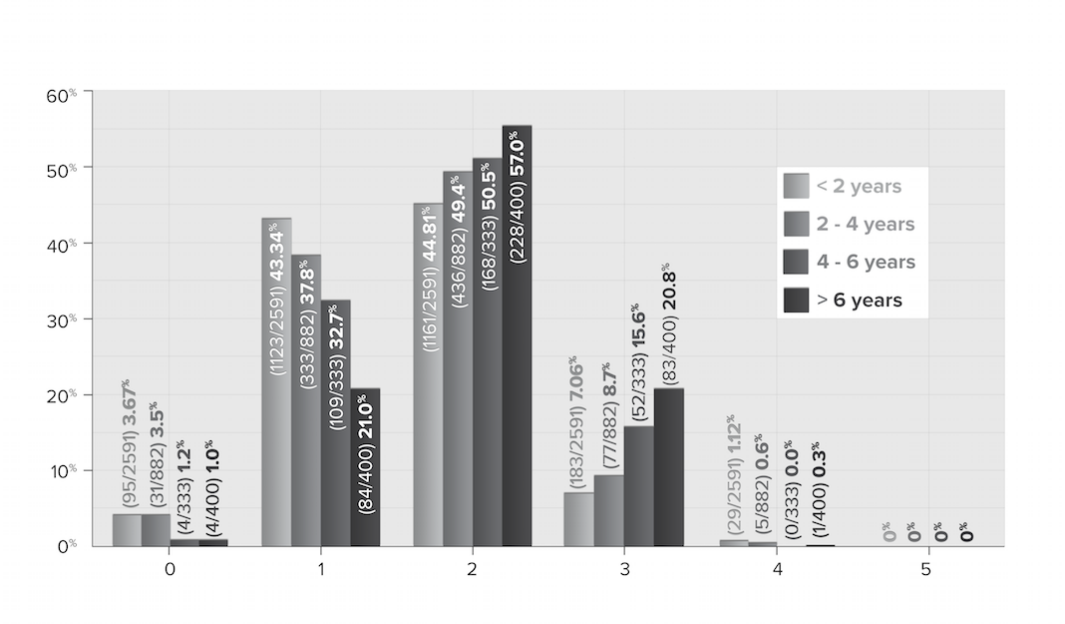
Maturity by years of use (N=4206).

**Figure 5:**
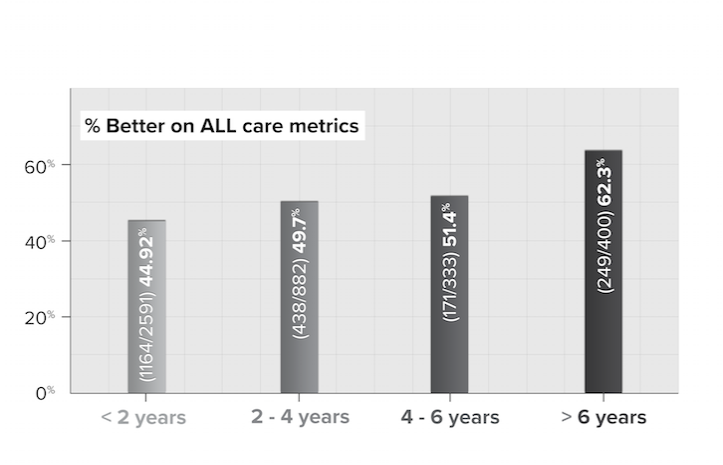
Percentage of physicians who indicate improvement on all patient care metrics by years of use (N=4206).

**Figure 6:**
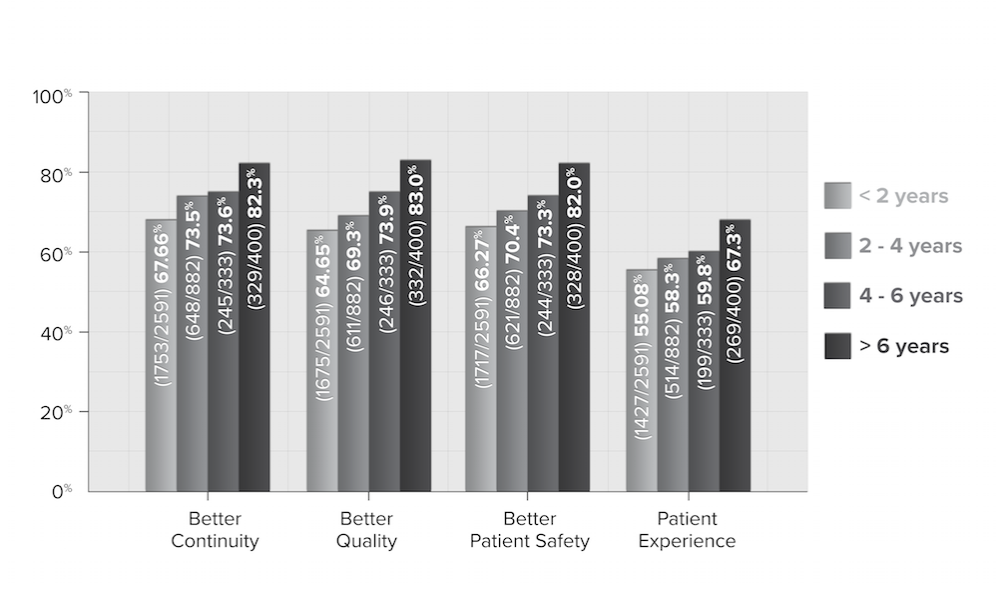
Physician perception across all patient care dimensions by years of use (N=4206).

Figure 4 shows physicians’ self-reported maturity level across increments of years of use. (As noted, each increment represents a different cohort, rather than the same cohort progressing across increments.) Results indicate movement through maturity levels over time (as one might expect, even given the limitations of this analysis). Of those responses completed at 4 or fewer years of use, 45.55% (1582/3473) reported an overall maturity of less than level 2, with an additional 45.98% (1597/3473) achieving an overall maturity of level 2, and only 8.47% (294/3473) progressing beyond level 2. In contrast, of those responses completed at more than 6 years of use, 57.0% (228/400) had reported an overall maturity level of 2, and 21.0% (84/400) had progressed beyond level 2. No respondents reported achieving level 5 on these functions. Overall results reflected that physicians are now integrating the EMR as an essential tool and using the core functionalities to engage patients in their day-to-day practice operation. For Figures 4-6, due to missing responses, the N is 4206 rather than 4214.

This snapshot of overall EMR maturity, although positive, masks the range of proficiency within each skill level as well as the impact of maturity on individual practices. Unraveling this aggregated information about multiple clinical functionalities could reveal a deeper understanding of issues that may limit mature EMR use. To this end, we selected 4 measures associated with patient care—continuity of care, quality of care, patient safety, and patient experience—to explore physicians’ perceptions of how EMRs affect their capability in these areas. These measures reflect Ontario practice and health system priorities of patient-centered care, as outlined in Health Quality Ontario’s primary care performance measurement framework [23] (informed by quality-driven frameworks such as Institute for Health Care Improvement’s Triple Aim) [24] and the Ontario government’s Patients First Action Plan [25], emphasizing sustainability through access to care, care coordination and integration, patient safety, and improved outcomes.

### Focus: Perception of Impact on Patient Care

The EPR asked physicians to rate their view of changes in their care approach since implementing an EMR if newly adopted, or over the last year if more experienced, on a 5-point scale: much worse, somewhat worse, about the same, somewhat better, or significantly better. The following 4 measures of patient care were addressed: quality of care, patient experience, continuity of care, and patient safety

Figure 5 shows the percentage of physicians whose response to that question was “somewhat better” or “significantly better” for all 4 measures. Physicians responding to the survey reported perceiving that the longer they used their EMR, the better patient care they provided. Those with at least two years of experience reported the greatest progress.

Furthermore, when the analysis was run for “same or better,” the percentages climbed by 10 for every increment. While it cannot be concluded from this data that longer EMR use translates into greater expertise—nor improved patient care—the correlation is suggestive and worth further exploration, especially as it is consistent with other findings [26]. As shown in Figure 6, patient care is perceived as continually improving, as physicians accumulate more EMR use over time.

## Discussion

While we are still accruing the longitudinal data to tell a more fulsome story about progress on enhanced use, our initial assessments suggest the following: (1) There is a direct correlation between years of EMR use and EMR maturity as measured in our model; (2) There is a similar positive correlation between years of EMR use and the perception that these systems improve clinical care in at least four patient-centered areas; and (3) There is evidence of ongoing improvement even in advanced years of use (ie, we have not yet plateaued on the benefit of change management efforts and practice improvement support).

Future analyses will have the benefit of insights from our peer leaders and EPEP field staff, in particular regarding the value of these change management strategies in supporting our enrolled clinicians in advancing to mature EMR use. However, as we gain these insights, we will also gain a risk of selection bias. Physicians who have invested time, money, and energy into the implementation of their EMR tool may feel frustration due to the disruption in their practice workflow inherent in large scale change, and slow progress in the first year or two of their use. After they have adapted to their new workflows, they may become more neutral or increasingly satisfied, tending to perceive—and report—their situation more positively (ie, “if I’ve stuck with my EMR this long I must be satisfied” versus “I’m very satisfied with a top notch product and its impact on my day”).

Nevertheless, we can conclude that there is utility in examining the interaction between an innovation, its intended adopters, and the particular context (here, community-based practice)—particularly in assisting the innovation’s spread and impact [27]. The change management approach used here recognizes that there are different types of adopters [22] whose needs and concerns vary. A strategy that studies differences between users and their workflow contexts, monitors their successes and obstacles, and assesses the value of supports (such as training focused on process and outcome rather than narrowly prescribed goals), can easily be adapted to other health technology challenges and contexts [28]. These factors were taken into account in the design of EPEP, where field staff supports physicians through site engagements, to address barriers, and optimize the value their EMR brings to their practice.

Whereas the findings presented here are based on EPR self-assessments completed from 2013-2016, we can expect to mine a richer collection of data going forward. We have already begun preliminary analyses of data collected with the new EPA tool and are supplementing this with quantitative and qualitative data from our teams in the field. From this we can expect to develop a more nuanced understanding of practice-level barriers to and facilitators of progress in enhanced use, which in turn will inform how our teams provide support to clinicians in order to sustain their progress—particularly as the digital health landscape evolves to realize better connectivity and access to a patient’s record at all points of care. The work required to maintain momentum, it is hoped, will be rewarded by observable (by clinicians) improvements in the quality of care delivered and in patient outcomes. It is clear from our experience that structured and measurable processes are critical to provide practices with effective ongoing support and training during and after EMR adoption. Our updated tools and approach will help us identify greater opportunities to help EMR users develop more sophisticated EMR capability, sustain and improve their proficiency, and build a more comprehensive view of the full potential of their EMR to benefit both their practice and the larger community of care.

## Abbreviations

ADKAR: awareness, desire, knowledge, ability, and reinforcement
EMR: electronic medical record
EMM: electronic medical record (EMR) maturity model
EPA: EMR progress assessment
EPR: EMR progress report
EPEP: EMR practice enhancement program
